# Chromosomal microarray analysis supplements exome sequencing to diagnose children with suspected inborn errors of immunity

**DOI:** 10.3389/fimmu.2023.1172004

**Published:** 2023-05-05

**Authors:** Breanna J. Beers, Morgan N. Similuk, Rajarshi Ghosh, Bryce A. Seifert, Leila Jamal, Michael Kamen, Michael R. Setzer, Colleen Jodarski, Rylee Duncan, Devin Hunt, Madison Mixer, Wenjia Cao, Weimin Bi, Daniel Veltri, Eric Karlins, Lingwen Zhang, Zhiwen Li, Andrew J. Oler, Kathleen Jevtich, Yunting Yu, Haley Hullfish, Bibiana Bielekova, Pamela Frischmeyer-Guerrerio, An Dang Do, Laryssa A. Huryn, Kenneth N. Olivier, Helen C. Su, Jonathan J. Lyons, Christa S. Zerbe, V. Koneti Rao, Michael D. Keller, Alexandra F. Freeman, Steven M. Holland, Luis M. Franco, Magdalena A. Walkiewicz, Jia Yan

**Affiliations:** ^1^ National Institute of Allergy and Infectious Diseases, National Institutes of Health, Bethesda, MD, United States; ^2^ National Cancer Institute, National Institutes of Health, Bethesda, MD, United States; ^3^ Baylor Genetics, Houston, TX, United States; ^4^ Department of Molecular and Human Genetics, Baylor College of Medicine, Houston, TX, United States; ^5^ Eunice Kennedy Shriver National Institute of Child Health and Human Development, National Institutes of Health, Bethesda, MD, United States; ^6^ National Eye Institute, National Institutes of Health, Bethesda, MD, United States; ^7^ Division of Pulmonary Diseases and Critical Care Medicine, School of Medicine, University of North Carolina at Chapel Hill, Chapel Hill, NC, United States; ^8^ National Institute of Arthritis and Musculoskeletal and Skin Diseases, National Institutes of Health, Bethesda, MD, United States

**Keywords:** genetic, copy number, immunity, sequencing, microarray, diagnosis, primary immunodeficiency, pediatric

## Abstract

**Purpose:**

Though copy number variants (CNVs) have been suggested to play a significant role in inborn errors of immunity (IEI), the precise nature of this role remains largely unexplored. We sought to determine the diagnostic contribution of CNVs using genome-wide chromosomal microarray analysis (CMA) in children with IEI.

**Methods:**

We performed exome sequencing (ES) and CMA for 332 unrelated pediatric probands referred for evaluation of IEI. The analysis included primary, secondary, and incidental findings.

**Results:**

Of the 332 probands, 134 (40.4%) received molecular diagnoses. Of these, 116/134 (86.6%) were diagnosed by ES alone. An additional 15/134 (11.2%) were diagnosed by CMA alone, including two likely *de novo* changes. Three (2.2%) participants had diagnostic molecular findings from both ES and CMA, including two compound heterozygotes and one participant with two distinct diagnoses. Half of the participants with CMA contribution to diagnosis had CNVs in at least one non-immune gene, highlighting the clinical complexity of these cases. Overall, CMA contributed to 18/134 diagnoses (13.4%), increasing the overall diagnostic yield by 15.5% beyond ES alone.

**Conclusion:**

Pairing ES and CMA can provide a comprehensive evaluation to clarify the complex factors that contribute to both immune and non-immune phenotypes. Such a combined approach to genetic testing helps untangle complex phenotypes, not only by clarifying the differential diagnosis, but in some cases by identifying multiple diagnoses contributing to the overall clinical presentation.

## Introduction

Inborn errors of immunity (IEI) include over 400 inherited disorders of the immune system with a wide spectrum of clinical manifestations (1). Patients may present with infections, autoimmunity, or risk of malignancy (2–7). While the symptoms of specific IEIs are wide-ranging, many IEIs have significant phenotypic overlap, making clinical diagnosis challenging (8).

Identifying the underlying genetic etiology of an IEI may have important implications for prognosis and treatment. A genetic diagnosis may impact management for roughly half of all patients diagnosed (5). For instance, molecular diagnosis may lead to the initiation of mechanism-based precision therapies or escalation to hematopoietic stem cell transplant (5, 9–11). From a counseling perspective, a genetic diagnosis can provide closure, clarify recurrence risk, and guide reproductive decision-making (11, 12).

Most IEIs are attributed to single-gene variants that cause abnormal immune function. Genetic testing strategies include targeted methods like Sanger sequencing, array-based platforms like chromosomal microarray analysis (CMA), and next-generation sequencing methods like targeted gene panels (TGPs) or exome/genome sequencing (ES/GS) (11, 13). While comprehensive approaches like ES and GS have relatively high diagnostic yield for IEI (14–18), cost, accessibility, and turnaround time can remain barriers (10). Our group previously completed ES analysis on 1000 families as part of an agnostic approach to exome analysis in IEI and found that 327/1000 probands received a molecular diagnosis (18). The degree to which CNVs contribute to diagnosis of IEI, particularly among children, has not been addressed in this cohort and remains an open question.

Certain CNVs are known to be significant in particular IEIs, most notably the 22q11.2 microdeletion implicated in DiGeorge syndrome and partial trisomy of 19p13 causing FURID19 (11). CNVs also affect other IEI-related genes, including *DOCK8* (19), *NCF1* (20), *FAS* (21), and many others (14). While the resolution of some CMA platforms may allow detection of CNVs as small as 10 kb (11), CMA is not designed to detect single nucleotide variants nor replace TGP or ES/GS as a sequencing platform. Rather, CMA is primarily useful as a supplement to these more granular sequencing approaches (10, 22). A 2020 expert opinion emphasized the importance of CNV analysis in the diagnosis of IEI and suggested that ES combined with CNV testing exhibits the best diagnostic yield for unexplained IEI (23). The complementary strengths and weaknesses of CMA and ES may be paired for a comprehensive evaluation in difficult-to-diagnose cases. Furthermore, some compound heterozygotes can only be diagnosed by combining the two tests (24).

While the impact of certain CNVs is well-characterized for specific IEIs (11, 14, 19, 20), the literature examining the overall role of CNVs in IEI is limited. Generally, the practice of molecular diagnostics related to CNVs is still maturing. Limited CNV frequency data cause a high number of apparently novel CNVs, making interpretation of these variants challenging. Interpretation of sequence variation received technical standards in 2015 (25); a similar framework was developed for CNVs only in 2020 (26).

The increase in diagnostic yield offered by CMA varies by cohort but is typically around 2-6% in both IEI and more general cohorts (14, 15, 23, 27, 28). However, literature exploring CNV contribution to diagnostic yield for IEI is scarce. In 2017, Stray-Pedersen et al. analyzed 278 families affected by IEI by performing ES and computational CNV analysis on all probands, followed by array validation of CNVs when predicted or when ES found no diagnosis. They found IEI-causing CNVs in 12/278 probands (4.31%), seven of whom were under 18 years old (14). Similarly, in 2022, Wan et al. specifically examined the contribution of CNVs to diagnostic yield in 191 patients with suspected IEI using computational methods to identify CNVs affecting immune-related genes based on ES data. They detected “clinically meaningful” CNVs in 2.6% (5/191) of patients across the lifespan (28). Both studies included both children and adults, used computational methods to predict CNVs prior to sending CMA, and only examined genes associated with immune conditions.

In general, early age of onset is associated with increased likelihood of a genetic contribution to disease, including contribution from CNVs (29, 30). However, further research is needed to elucidate the association between CNVs and age of onset for IEI, as well as the potential impact of CNVs on clinical presentation.

Noting that CNVs play a significant but largely unexplored role in IEI etiology and that genetic disease may be more common with earlier age of onset, we sought to assess the contribution of CMA to diagnostic yield in children with IEI. We performed ES and CMA for pediatric participants with IEI as part of a larger study with long-term research goals exploring variable expressivity and reduced penetrance within known disease categories. Because commercial CMA platforms are mostly geared towards genes involved in neurodevelopmental phenotypes, we employed a custom oligonucleotide microarray that offered coverage across the genome and high-resolution exon-level coverage of genes known or suspected to be involved in human immunity. This clinical genome-wide array coverage distinguishes our study from prior analyses that used computational methods to focus on CNVs in known immune genes. In the context of our research question, we analyzed a narrow subset of our previously published research cohort (18) along with 128 new pediatric participants to directly assess the role of CNVs in IEI in children.

## Methods

Participants with clinically established IEI were referred to the National Institute of Allergy and Infectious Diseases (NIAID) Centralized Sequencing Program for clinical research evaluation from 2017 to 2021. Written informed consent was obtained for all participants and the study was approved by the National Institutes of Health (NIH) Institutional Review Board (NCT03206099). We performed ES for all participants. CMA was performed for a subset selected based on various factors, including (1) clinical phenotypes, prioritizing those with syndromic features; (2) findings from ES data, such as a monoallelic variant in a gene for an autosomal recessive disorder; and (3) agnostically after receiving inconclusive ES results. We report the results of 332 unrelated probands under 18 years old who received both ES and CMA.

### Exome sequencing

Research-based ES was performed as described in **Supplementary Methods**. Potentially relevant variants were confirmed by Sanger sequencing or other appropriate methods meeting Clinical Laboratory Improvement Amendments/College of American Pathologists (CLIA/CAP) requirements. ES data was used to estimate the degree of consanguinity within the cohort, using absence of heterozygosity (AOH) to calculate percent identity by descent (IBD). Additional details are available in **Supplementary Methods.**

### Chromosomal microarray

A custom 180,000-oligonucleotide comparative genomic hybridization microarray was designed in collaboration with Agilent Technologies (Santa Clara, CA). Backbone genome coverage aimed for 30 kb resolution in intergenic regions, 10 kb resolution in intronic regions, and single-exon resolution in a set of 2,408 genes known or suspected to be involved in human immunity. More than 99% of the targeted exons were covered on or near the coding sequence by at least 3 oligonucleotide probes. Additionally, Participant S1403674 received a second CMA of a more comprehensive design that included a single nucleotide polymorphism microarray, allowing for the technical feasibility necessary to clinically confirm a research finding of uniparental disomy. Additional details on both arrays can be found in **Supplementary Methods**.

### Phenotypic analysis and variant interpretation

Standardized detailed phenotyping was performed using the Human Phenotype Ontology (HPO) in PhenoTips (31, 32). Sequence variants were interpreted in accordance with the American College of Medical Genetics and Genomics (ACMG) and Association for Molecular Pathology technical standards (25). Results returned to participants included primary findings related to participants' clinical phenotypes, secondary findings recommended by the ACMG (33, 34), and select incidental findings that may not have been suspected clinically, but for which genetic evidence strongly supported pathogenicity. Copy number variants were analyzed at Baylor Genetics using their internal database and CLIA/CAP protocol (35).

Molecular diagnoses were determined as follows:

1. A single pathogenic or likely pathogenic variant for disorders that follow autosomal dominant inheritance in either sex or X-linked inheritance in males; or
2. Two pathogenic or likely pathogenic variants in the same gene for disorders that follow autosomal recessive inheritance where segregation data were unavailable or suggested a *trans* configuration; or
3. A single pathogenic or likely pathogenic variant and a variant of uncertain significance (VUS) in the same gene for disorders that follow autosomal recessive inheritance where segregation data were unavailable or suggested a *trans* configuration.

All cases were reanalyzed in light of the most recent International Union of Immunological Societies (IUIS) gene list released in 2022 (36). We reviewed variants in genes added to the IUIS list that were not in Human Gene Mutation Database (HGMD) at the time of analysis but have since been reported in the literature (37). Additionally, we reviewed CNVs called using GATK-SV (38) in 11 participants who received follow-up genome sequencing, with a particular focus on CNVs in new IUIS genes that could have been missed by prior CMA.

### Statistical analysis

Phenotypic correlates of molecular diagnoses were assessed using ANOVA, t-tests, and Fisher’s exact tests. We generated genomic ancestry principal components from germline variation using peddy version 0.4.6 (39) and the 1000 Genomes phase 3 reference panel of 2,504 individuals (40), and principal components 1, 2, and 3 were plotted using the ggplot2 package in R (41). Statistical analyses were performed using R version 3.5.3 (41).

## Results

### Cohort characteristics

**Figure 1:**
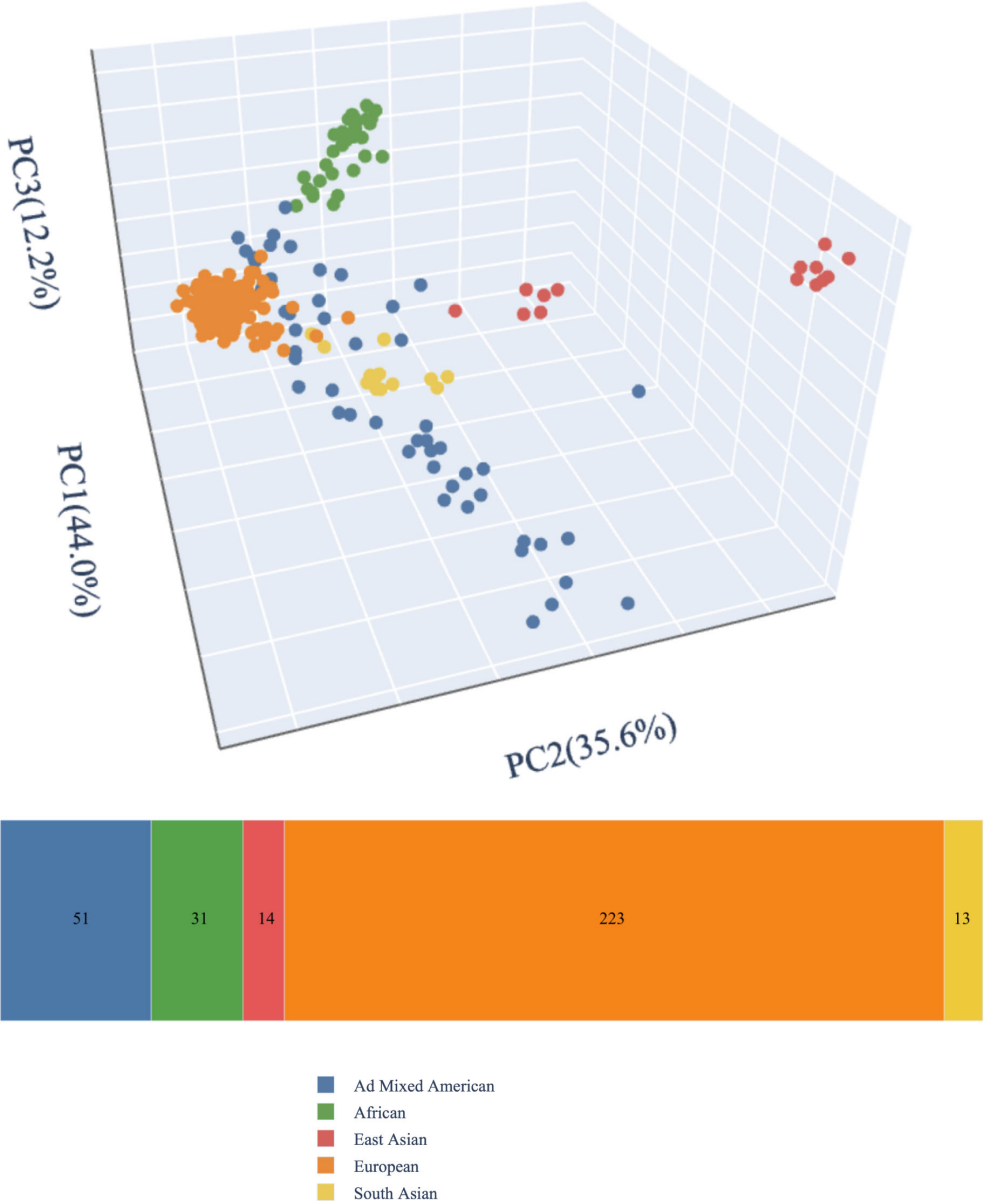
Genetic ancestry. We generated genomic ancestry principal components from germline variation using peddy version 0.4.6 (39) and the 1000 Genomes phase 3 reference panel of 2,504 individuals (40).

Our cohort included 332 probands under 18 years old at the time of enrollment (*M* = 9.9 years, *SD* = 4.3 years). Participants were 56.6% male. Participants’ self-reported race and ethnicity are reported in **Table 1**; inferred genetic ancestry from ES data is mapped in **Figure 1**. The cohort was predominantly non-consanguineous. Five participants (1.5%) had percent IBD estimates >6.25%, indicating offspring of first cousins or closer relations (42); none of these were diagnosed by CMA. An additional six participants (1.8%) had percent IBD estimates between 1.56% and 6.25%, indicating offspring of second cousins or closer relationships up to that of first cousins; of these, three individuals were diagnosed by CMA only. There was no significant difference in proportion of individuals with diagnoses from CMA only, diagnoses from ES only, and no molecular diagnoses based on inferred genetic ancestry (*p* = 0.36 [Fisher’s exact test]), sex (*p* = 0.11 [Fisher’s exact test]), age (*F*(2, 325) = 1.83, *p* = 0.16), or percent IBD (*F*(2, 325) = 2.37, *p* = 0.096). Approximately one-quarter (82/332, 24.7%) of participants had at least one established molecular diagnosis from previous genetic testing at the time of enrollment, though further updated analyses were warranted to support research and clinical goals.

**Table 1:** Demographic characteristics of cohort, including age at enrollment, sex, and self-reported race and ethnicity.

Demographics	N	%
Age at enrollment		
0 - 5 years	58	17.5
6 - 9 years	85	25.6
10 - 13 years	110	33.1
14 - 17 years	79	23.8
Sex		
Female	144	43.4
Male	188	56.6
Race		
American Indian or Alaska Native	5	1.5
Asian	21	6.3
Black or African American	24	7.2
Native Hawaiian or Pacific Islander	1	0.3
White	249	75
Multiple races	23	6.9
Unknown	9	2.7
Ethnicity		
Hispanic or Latino	40	12.1
Not Hispanic or Latino	274	82.5
Unknown	18	5.4

About one-third of participants (107/332, 32.2%) had phenotypes involving ten or more top-level HPO categories, roughly corresponding to organ systems. In order of decreasing frequency, the five most common top-level HPO categories were the immune system, integument, respiratory system, digestive system, and blood and blood-forming tissues.

### CMA contribution to diagnostic yield

**Figure 2:**
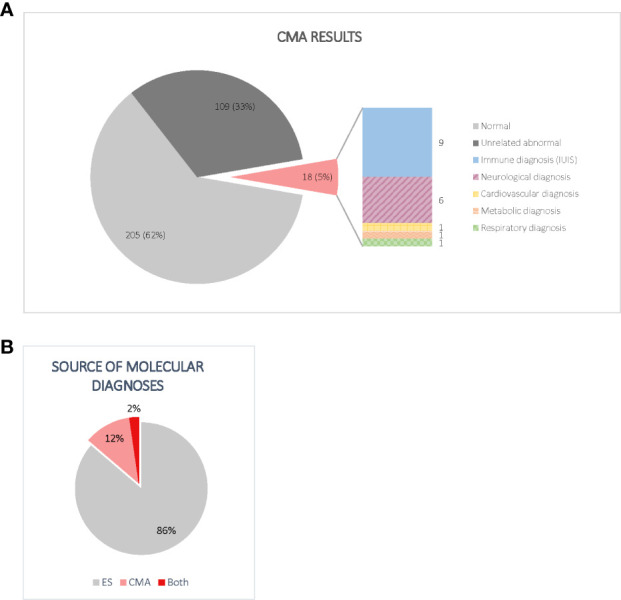
**(A)** Distribution of CMA results. Normal CMAs included all tests that did not find evidence of CNVs meeting the performing laboratory’s reporting guidelines. Non-diagnostic abnormal CMAs included CNVs of unclear clinical significance, findings of carrier status only, and changes in non-disease associated regions. Importantly, of the 18 diagnostic CNVs, only half included genes listed by the IUIS as a cause of IEI at the time of analysis. **(B)** Source of molecular diagnoses. Although most participants were diagnosed by ES alone, CMA accounted for a significant increase in diagnostic yield. Most participants with diagnostic CNVs had inconclusive ES results.

Of the 332 CMAs performed, 205/332 (61.7%) did not identify any clinically significant CNVs. Another 109/332 (32.8%) consisted of abnormal CMA results that were considered unrelated to the proband’s condition for a variety of reasons: 53/109 (48.6%) found CNVs of unclear clinical significance, 30/109 (27.5%) identified CNVs in non-disease-associated regions, 20/109 (18.3%) were findings of carrier status only, and 6/109 (5.5%) were deemed unrelated for other reasons. Ultimately, 18/332 participants (5.4%) had a CMA that detected abnormal findings that contributed to a molecular diagnosis, as shown in **Figure 2A**.

When we combined the results from both ES and CMA, 59.6% of participants (198/332) did not receive a molecular diagnosis. The methods that detected diagnostic variants in the 134/332 (40.4%) participants who received a molecular diagnosis are visualized in **Figure 2B**. Of these, 116/134 (86.6%) received a molecular diagnosis from ES alone, while 15/134 (11.2%) received a molecular diagnosis from CMA alone. Three individuals (2.2%) received molecular diagnoses from both ES and CMA combined, including two compound heterozygotes and one participant with two distinct diagnoses. The primary genes and genomic regions implicated in the 18 participants with CMA contribution to diagnosis are listed in **Table 2**. Reanalysis considering the 2022 update to the IUIS gene list did not result in any new CMA diagnoses or change any existing diagnoses. Overall, performing CMA raised the diagnostic yield from 34.9% (116/332) to 40.4% (134/332), a 15.5% increase.

**Table 2:** CNVs implicated in diagnoses by CMA.

Participant code	Sex	Age	CMA finding	Minimum interval	Size (kb)	Gene of interest	IUIS gene?	Inheritance pattern	Zygosity	Inheritance finding
S6911406	M	5	1p36.23p36.22 gain	(8231915–9780171) x3	1548.256	*PIK3CD*	Yes	AD	Het	*-*
S2442477	M	6	1q42.3q43 loss	(234747397–239456926) x1	4709.529	*IRF2BP2*	Yes	AD	Het	*De novo*
S3780156	M	7	2q33.2q33.3 loss	(204169244–207244380) x1	3075.136	*CTLA4*	Yes	AD	Het	-
S4796685	F	15	5p15.2 loss	(13922477-13923557) x1	1.08	*DNAH5*	No	AR	Comp. het	–
S2258929	F	13	9p24.3 loss	(210253-317136) x1	106.883	*DOCK8*	Yes	AR	Comp. het	-
S3014542	M	7	9p24.3 loss	(311862-340315) x0	28.453	*DOCK8*	Yes	AR	Hom	Paternal†
S5334530	M	10	9p24.3 loss	(211086-703693) x0	492.607	*DOCK8*	Yes	AR	Hom	Paternal UPD
S4896433	M	2	9p24.3q21.11 gain	(115981-70901873) x4	70785.892	NA	No	NA	Hom*	–
S8763051	F	2	10p13 loss	(14987006-14996917) x0	9.911	*DCLRE1C*	Yes	AR	Hom	-
S6703475	M	8	10q23.31 loss	(89851273-90855104) x1	1003.831	*FAS*	Yes	AD	Het	Maternal
S1308487	M	11	10q23.31 loss	(90771628-90815977) x1	44.349	*FAS*	Yes	AD	Het	Paternal
S4939706	M	16	12q12 loss	(46189647-46339053) x1	149.406	*ARID2*	No	AD	Het	–
S5696835	M	11	15q22.33 loss	(67473997-67481381) x1	7.384	*SMAD3*	No	AD	Het	Paternal†
S0600549	M	9	16p11.2 gain	(29673956-30199664) x3	525.708	NA	No	AD	Het	–
S1403674	M	10	16p11.2 gain	(28493787-28495303) x4	1.516	*CLN3*	No	AR	Hom	Maternal UPD
S2174752	F	4	17p12 gain	(14111772-15442069) x3	1330.297	CMT1A region	No	AD	Het	*De novo*
S4154908	F	10	Xp21.1p11.4 loss	(34551214-38352080)x1	3800.866	*CYBB, OTC*	Yes, no	XL	Het	-
S3277789	M	14	Xp21.1p11.4 loss	(37473737-37644155) x0	170.418	*CYBB, XK*	Yes, no	XL	Hem	–

*Possible mosaicism; †mother not tested; AD, autosomal dominant; AR, autosomal recessive; XL, X-linked; Het, heterozygous; Hom, homozygous; Comp. het, compound heterozygous; Hem, hemizygous; UPD, uniparental disomy.

### Characteristics of diagnostic copy number variants

As shown in **Figure 2A**, 9/18 (50.0%) participants who received CMA diagnoses had variants in at least one gene not listed by the IUIS as a cause of an IEI at the time of analysis. Of the nine participants with non-IUIS molecular diagnoses, six were primarily neurological (*ARID2-*related Coffin-Siris syndrome, Charcot-Marie-Tooth Disease Type 1A, *CLN3*-related Batten disease, tetrasomy 9p, *XK-*associated McLeod syndrome, and 16p11.2 duplication), one was a connective tissue disorder (*SMAD3-*related Loeys-Dietz syndrome), one was metabolic (*OTC*-related ornithine transcarbamylase deficiency), and one was respiratory (*DNAH5-*related primary ciliary dyskinesia).

**Figure 3:**
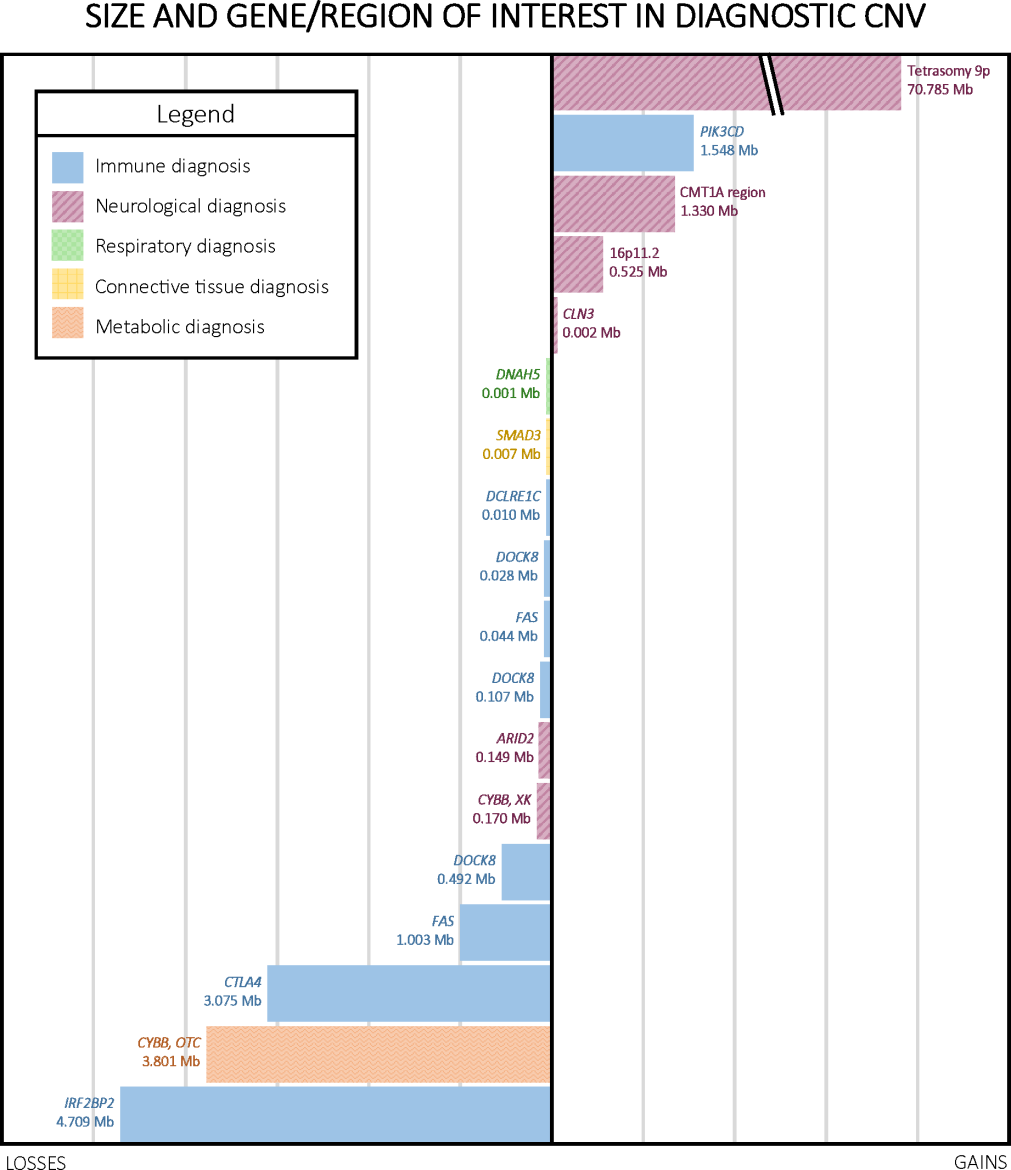
Size, type, and region of interest in diagnostic CNVs. CNVs ranged in size from 1.08 kb to 70.786 Mb, with a median of 0.493 Mb. Notably, four out of the five copy number gains in our cohort were linked to neurological phenotypes.

Diagnostic CNVs ranged in size from 1.08 kb to 70.786 Mb, with a median of 0.493 Mb. The size distribution of diagnostic CNVs is illustrated in **Figure 3**. Interestingly, 6/18 (33.3%) participants with diagnostic CNVs also had at least one additional, non-diagnostic copy number VUS. Of the participants without diagnostic CNVs, 18/109 (16.5%) had multiple CNVs reported as VUS. However, the difference in presence of multiple CNVs reported as VUS among individuals with diagnostic CNVs compared with individuals who had only non-diagnostic CNVs was not statistically significant (*p* = 0.11 [Fisher’s exact test]). A complete list of all CNVs reported can be found in **Supplementary Table 1**.

Of the 18 diagnoses made by CMA, nine were for disorders that typically follow an autosomal dominant inheritance pattern, six were for autosomal recessive disorders, two were X-linked, and one was an error of early embryonic development not typically inherited (tetrasomy 9p). Parental CMA was unavailable for ten of these participants, but the remaining eight had at least one parent studied by CMA, which was used to infer the *cis* or *trans* configuration of CNVs observed in the proband. Parental CMA confirmed paternal or maternal inheritance of CMA findings for six of these eight participants. One participant’s homozygous copy number gain interrupting the *CLN3* gene resulted from maternal uniparental disomy of a duplicated region. Another participant’s homozygous deletion of *DOCK8* arose from segmental paternal uniparental disomy. Additionally, negative parental CMAs for two probands indicated that the CNVs likely arose *de novo* in the probands, both of which were heterozygous copy number gains, one encompassing *IRF2BP2* and the other including the Charcot-Marie-Tooth Type 1A region (**Table 2**). We did not observe a significant difference in parental age between *de novo* and inherited diagnostic CNVs.

## Discussion

Using ES and CMA, we identified or confirmed a molecular diagnosis in 134/332 pediatric participants (40.4%) with IEI. CNVs contributed to 18/134 diagnoses (13.4%), increasing the overall diagnostic yield by 15.5% beyond ES alone. Non-diagnostic CNVs were found in one-third of all probands (109/332, 32.8%). Notably, half the participants who received CMA diagnoses had diagnostic variants in at least one gene not currently listed by the IUIS as the cause of an IEI, highlighting the importance of comprehensive genotypic and phenotypic evaluation of patients with IEI; including only known immune genes in CNV analysis would have reduced CMA contribution to diagnostic yield by half. The complexity of these cases is further emphasized by data indicating that nearly one-third of participants (107/332, 32.2%) had phenotypes involving ten or more top-level HPO categories, roughly corresponding to organ systems.

The diagnostic yield of CNV analysis varies based on cohort characteristics (15, 23). Studies examining the contribution of CNVs to diagnostic yield for IEI also vary (14, 15), which may be explained by different targets for computational methods calling CNVs in immune genes from ES data and the specific characteristics of different research cohorts. Unlike prior studies in IEI cohorts, we used clinical CMA rather than computational methods to call CNVs across the genome and focused our analyses on pediatric participants. This study expanded on our prior work studying the contribution of ES to IEI evaluation in both children and adults with the addition of 128 new participants and 8 additional diagnoses. Specifically, this design allowed us to assess the role of CNVs in young participants using clinical CMA, whereas our prior work focused on ES findings regardless of age and did not directly evaluate the role of CNVs. Our finding of a 15.5% increase in diagnostic yield from CMA is comparable to but slightly higher than other studies (14, 23, 28). To our knowledge, this is the first study specifically examining the diagnostic yield of CMA in pediatric participants with IEI.

The reasons for performing CMA in the participants we describe were varied; some were based on clinical phenotypes and/or ES findings of a single allele for an autosomal recessive disorder, while others were sent agnostically following inconclusive ES. As a result, we predict that the diagnostic contribution of CMA would be lower if this test were performed in an exclusively agnostic manner. Conversely, we would expect the diagnostic contribution of CMA to be greater when CMA is conducted on an exclusively targeted basis.

Fifty-three CMAs returned only copy number VUS. While VUS may be reclassified over time as scientific understanding of the human genome increases, the practical logistics of reclassification at a large scale may limit implementation in practice. Genetic counseling serves an important role in contextualizing these findings for both patients and providers, since misinterpretation of these variants can lead to unnecessary emotional distress or medical mismanagement (15).

Parental CMA was performed for 8/18 participants with diagnostic CNVs. Although three diagnostic CNVs in genes associated with autosomal dominant disorders were parentally inherited (**Table 2**), parents were not affected, consistent with prior reports of reduced penetrance in these genes (21, 43). Two (25.0%) participants’ CNVs apparently arose *de novo.* These diagnoses highlight the importance of inheritance testing for genetic counseling, as they imply a low recurrence risk for these parents to have another affected child. Familial testing may also help inform variant interpretation for copy number VUS.

At least one gene not associated with an IEI was implicated in half of the participants who received diagnoses from CMA (9/18, 50.0%). In some cases, these non-IUIS findings explained the participant’s immunological phenotype. For instance, a known 1 kb deletion was identified in *trans* with a pathogenic nonsense variant in *DNAH5*. Recessive loss of function variants in *DNAH5* are associated with primary ciliary dyskinesia (PCD). While the root cause of the associated phenotype lies outside the immune system, PCD manifests with several of the same symptoms as many IEIs, including recurrent bacterial respiratory infections, chronic cough, and bronchiectasis. Similarly, while *TGFBR1-* and *TGFBR2-*related Loeys-Dietz syndrome are included in the IUIS gene list for IEI, *SMAD3-*related Loeys-Dietz syndrome is not, despite manifesting with many of the same symptoms. Given the complexity of IEI phenotypes, we were not surprised to find relevant variants outside the current list of IEI-associated genes when performing comprehensive genetic evaluation. These cases highlight the value of a molecular diagnosis in clarifying the root cause of conditions that may be clinically indistinguishable from one another and illustrate the limitations of relying on narrow analysis.

In other cases, non-IUIS findings contributed to a condition unrelated to the participant’s immunological phenotype. None of these CNVs involved genes listed as secondary findings by the ACMG; instead, they explained specific components of the participants’ phenotypes, even when they did not provide answers for the primary reason for referral. The majority (6/9) of the clinically significant non-immunological CNVs were neurological diagnoses. For example, Participant S2174752 presented with coccidioidomycosis and various neurological symptoms that were presumed related to infection of the brain and spine. However, CMA showed a CNV causative of Charcot-Marie-Tooth Disease Type 1A. This rare nervous system disorder can lead to weakness, atrophy, and sensory loss in the limbs beginning in adolescence. This CMA finding was found to be consistent with and more likely to explain the participant’s neurological phenotype. This case demonstrates the potential for unexpected diagnoses that comprehensive genetic evaluation can provide. Particularly for patients with multiple diagnoses, a common occurrence in IEI clinical practice, it can be difficult to distinguish the root cause of a given phenotype, and careful consideration is required for the return of results and clinical decision-making.

More generally, the high incidence of non-IUIS diagnostic CNVs emphasizes the importance of a comprehensive approach including both genotypic and phenotypic evaluation in patients with IEI. Nearly one-third of participants’ phenotypes involved ten or more top-level HPO categories. It can be difficult to rule out the possibility of multiple diagnoses without comprehensive evaluation. Further, since immune dysfunction can impact every organ system, these complex cases can expand our understanding of the phenotypic spectrum in rare immune conditions.

In some cases, a contiguous gene deletion that encompassed multiple genes contributed to a complex phenotype. For instance, Participant S4154908 presented with recurrent respiratory infections, nausea and vomiting, low circulating ornithine levels, and global developmental delay. CMA identified a 3.801 Mb heterozygous deletion on Xp21.1p11.4 affecting 17 genes, including *CYBB* and *OTC*. Variants in *CYBB* are associated with X-linked chronic granulomatous disease (CGD) and immunodeficiency 34. While *CYBB-*associated conditions are classified as X-linked recessive, female carriers, like this participant, often exhibit less severe immune phenotypes, including recurrent infections and gastrointestinal inflammation. Similarly, female heterozygotes carrying defects in *OTC* have been known to manifest symptoms of ornithine transcarbamylase deficiency under metabolic stress. This case highlights that diagnostic CNVs may involve multiple genes in combination that contribute to a participant’s overall phenotype, as opposed to sequence variants that primarily manifest as monogenic disorders. These patients may be clinically complicated and require heightened attention to medical management.

In other cases, multiple types of variants contribute to a single genetic diagnosis, necessitating combined evaluation of data from multiple types of genetic testing. As discussed above, in Participant S4796685, ES identified a heterozygous c.6763C>T (p.Arg2255Ter) nonsense variant in *DNAH5* and subsequent CMA detected a 1 kb copy number loss affecting *DNAH5*, thus explaining the participant’s phenotype and establishing the molecular diagnosis of PCD, an autosomal recessive disorder. Cases like this that require multiple tests to reach a diagnosis highlight the utility of combining ES and CMA for comprehensive analysis.

Consistent with previous studies (14), nearly two-thirds of participants with clinical features suggestive of IEI did not receive a molecular diagnosis, highlighting the need for a greater understanding of the complex factors that can lead to IEI, including mosaicism, genetic modifiers, epigenetic regulation, environmental factors, and the stochastic nature of rearrangements leading to the repertoire of B and T cell receptors in each individual (28, 44, 45). ES data does not allow consistent detection of CNVs due to lack of coverage across non-coding regions; due to these technical limitations, we opted for clinical CMA rather than *in silico* methods to call CNVs, ensuring the validity of our results for return to research participants. The relevance of CMA in identifying CNVs is evolving with the increasing ability to detect CNVs from genome sequencing (GS) data. However, at this time, GS remains an inaccessible option for many patients outside of a research setting, the ability of the predominant short-read sequencing methodologies to identify structural variants seems to be incomplete (38), and direct comparisons of the clinical performance of array-based versus GS-based CNV analysis in IEI have not been performed at scale.

This study has several limitations. First, our participants are a research cohort with extensive prior workup, which may select for particularly complicated cases of undiagnosed IEI. For instance, some classic IEIs such as complement deficiency were underrepresented or absent. Thus, we expect that the diagnostic yield may be higher among pediatric patients being evaluated for IEI for the first time, since cases that haven’t had extensive prior inconclusive genetic testing may be more likely to yield molecular diagnoses upon initial evaluation. Second, as discussed above, the varied reasons for CMA may affect the diagnostic yield relative to cohorts sending CMA on a wholly agnostic or more selective basis, as opposed to the combined approach described above. Previous molecular diagnoses also informed the decision to perform CMA for some participants, although our comprehensive workup identified additional variants of interest in some participants referred with an initial diagnosis. Finally, although our CMA was designed to detect CNVs in IUIS genes, not every exon could be covered with an oligonucleotide probe due to technical limitations; small CNVs in these regions may have been missed. The design also did not take into consideration the recent update to the IUIS genes list. To address these issues, we are now using GS data to detect CNVs.

Molecular diagnosis of IEI can be complex due to the potential for many overlapping factors contributing to patient phenotypes. Establishing a molecular diagnosis has substantial implications for medical management and genetic counseling. We describe the molecular diagnostic contribution of CMA as a supplement to ES in children with IEI, highlighting the role of CNV detection in the diagnosis of IEI. CMA contributed to 13.4% of all diagnoses in this cohort, a 15.5% increase in diagnostic yield. Notably, half of CMA diagnoses at least partially involved non-immune genes, which would not typically appear on commercial panels for IEI. We observed that this two-pronged approach to genetic testing helped untangle complex phenotypes, not only by clarifying the differential diagnosis, but, in some cases, by identifying multiple diagnoses that contributed to the participant’s overall presentation. For children with unexplained IEI, coupling CMA and ES can provide a comprehensive evaluation that clarifies the complex factors contributing to both immune and non-immune phenotypes.

## Data availability statement

The datasets presented in this study can be found in online repositories. The names of the repository/repositories and accession number(s) can be found below: phs001899.v2.p1 (dbGaP).

## Ethics statement

The studies involving human participants were reviewed and approved by National Institutes of Health Institutional Review Board (NCT03206099). Written informed consent to participate in this study was provided by the participants’ legal guardian/next of kin.

## Author contributions

All authors made substantial contributions to the work as outlined by the International Committee of Medical Journal Editors (ICMJE), including the conception or design of the work and contributing to drafting and revising the work critically for important intellectual content. BJB and JY collected the data for the study and drafted the manuscript. MS, MAW, BAS, and RG made substantial contributions to data interpretation and manuscript revisions. EK, DV, LZ, and ZL contributed to reanalysis of data and interpretation of findings. AJO calculated identity by descent estimates based on absence of heterozygosity. WC, JY, and BJB created figures. WB, LJ, MK, MRS, CJ, RD, DH, MM, KJ, YY, and HH contributed to data collection and provided approval of the work. SMH, LMF, BB, PF-G, AD, LAH, KNO, HCS, JJL, CSZ, VKR, MDK, and AFF provided critical expertise and revised the manuscript for important intellectual content. All authors provided final approval of the manuscript and agree to be accountable for all aspects of the work.
