# Supplementary material for: Chromosomal microarray analysis supplements exome sequencing to diagnose children with suspected inborn errors of immunity
Source: Front Immunol. 2023 May 5;14:1172004. doi: 10.3389/fimmu.2023.1172004 (PMC10196392; doi:10.3389/fimmu.2023.1172004)
Supplement: Supplementary file 1 [file DataSheet_1.docx]

**Chromosomal Microarray Analysis Supplements Exome Sequencing to Diagnose Children with Suspected Inborn Errors of Immunity**

Breanna Beers, Morgan Similuk, Rajarshi Ghosh, Bryce A. Seifert, Leila Jamal, Michael Kamen, Michael R. Setzer, Colleen Jodarski, Rylee Duncan, Devin Hunt, Madison Mixer, Wenjia Cao, Weimin Bi, Daniel Veltri, Eric Karlins, Lingwen Zhang, Zhiwen Li, Andrew Oler, Kathleen Jevtich, Yunting Yu, Haley Hullfish, Bibi Bielekova, Pamela Frischmeyer-Guerrerio, An Dang Do, Laryssa A. Huryn, Kenneth N. Olivier, Helen C. Su, Jonathan J. Lyons, Christa S. Zerbe, V. Koneti Rao, Michael D. Keller, Alexandra F. Freeman, Steven M. Holland, Luis M. Franco, Magdalena A. Walkiewicz, Jia Yan*

*Frontiers in Immunology*

Corresponding author: [Jia](mailto:Jia) Yan, jia.yan@nih.gov

This resource contains supplementary material regarding methodology of exome sequencing, absence of heterozygosity calculations, and chromosomal microarray design.

**Exome sequencing**

Research-based exome sequencing was performed at the Center for Inherited Disease Research (CIDR) at Johns Hopkins University and the Human Genome Research Center (HGSC) at Baylor College of Medicine on study participants with an available sample. Quality control included electrophoresis, UV absorbance readings and volume checks that were performed upon sample receipt at CIDR and HGSC to confirm adequate quantity and quality of genomic DNA. Prior to sequencing, samples were processed with the Illumina HumanCoreExome-24v1-2 array or the Infinium Global Screening Array 24v2-v3 to confirm sex, relationship, unexpected duplicates, cryptic relatedness, and provide sample performance information and sample identity confirmation against the sequencing data. The exome capture was performed using the Agilent SureSelect XT HumanAllExon V6+UTR 91 Mb kit S07604624. Libraries were sequenced on the Illumina HiSeq2500 platform using 125 bp paired end runs or the NovaSeq 6000 platform using 100 bp paired end runs. Intensity analysis and base calling was performed through the Illumina Real Time Analysis (RTA) software (version 1.17.20). Basecall files were demultiplexed from a binary format (BCL) to single sample fastq files using a demultiplexer written at CIDR as part of CIDRSeqSuite version 6.1 (unpublished). Fastq files were aligned with BWA-MEM version 0.7.8 to the 1000 genomes phase 2 (GRCh37) human genome reference. Picard versions 1.109 and 2.17.0 were used to flag duplicate molecules. Local realignment around indels and base call quality score recalibration and binning (2,10,20,30) were performed using the Genome Analysis Toolkit (GATK)(E1) version v4.0.1.1. Cram files were generated using SAMTools version 1.7. GATK’s reference confidence model workflow was used to perform joint sample genotyping using GATK version 3.7. Variant filtering was completed using the Variant Quality Score Recalibration (VQSR) method [21478889](E2). All variants in the final multi-sample VCF file are annotated using Annovar (version 2013_02_21) against a variety of data sources including gene annotation, function prediction and frequency information. Summary statistics (for SNVs and INDELs) on the multi-sample .vcf file are calculated for each variant (both PASS and FAIL) including counts and frequencies of alleles and genotypes, missing rates, overall quality scores, and mean depth.

**Absence of heterozygosity**

Regions of Absence of Heterozygosity (AOH) were determined by first calling runs of AOH on autosomes using ‘bcftools roh’, ‘gemini roh’, and ‘plink --homozyg’(E1–3). For each method, variants were first filtered using bcftools to retain mono-allelic, single nucleotide variants with no missing alleles that passed VQSR (at 99.9% sensitivity) and had allelic balance ≥ 0.25 and genotype quality ≥ 20. For bcftools roh, we used default settings, along with gnomad exomes as the allele frequency database. For gemini roh we used a minimum genotype depth of 10 and otherwise kept default settings. For plink, we first filtered for mean depth ≥ 10 and converted the VCF to ped + map format using vcftools (E4) and then ran plink --homozyg using default settings. We used ‘bedtools multiinter’(E5) to determine regions overlapping between at least two methods, which were considered the consensus ROH an individual. Percent identity by descent (IBD) estimates were calculated based on the sum of autosomal AOH segments of at least 3Mb divided by the autosomal genomic length.

**Chromosomal microarray**

To analyze copy number variation in immune-related genes and regions at the individual exon level, a custom comparative genome hybridization (CGH) array was designed in collaboration with Agilent Technologies (Santa Clara, CA) and performed at Baylor College of Medicine under test code 8658. The following immune system-related targets were included in this 4x180K custom design: 1) 2,408 genes known or predicted to be involved in immunity (Gene Ontology terms containing immun*) were targeted for single-exon resolution (3-4 probes per gene); 2) noncoding exons from 15 RNA genes or pseudogenes; 3) three genomic regions of special interest. Design parameters included using the hg19 genome build and avoiding known segmental duplications. Overall, more than 99% of the targeted exons are covered by at least 3-4 probes, each of the 2,408 genes had one probe approximately every 10 kb, and the entire genome backbone had one probe approximately every 55 kb. Therefore, copy number variants could be evaluated on individual exon level or gene level (for targeted genes) and genome-wide with lower resolution. The design has a design ID 085277 in the Agilent system and its covered.bed file is available upon request.

Additionally, P0007919 received a second chromosomal microarray of a more comprehensive design in order to detect a previously known molecular diagnosis. This array was designed in collaboration with Agilent Technologies (Santa Clara, CA) performed at Baylor College of Medicine under test code 8665. The design included high resolution (HR) copy number analysis and single nucleotide polymorphisms (SNP) for the detection of absence of heterozygosity (AOH) and uniparental disomy (UPD). Benefits include exon-by-exon coverage of over 4,200 clinically significant genes, whole genome backbone coverage at a 30 Kb resolution, tiling coverage of mitochondrial genome, and 57,000 oligos used for detection of AOH associated with UPD or consanguinity. AOH less than 10 Mb in size were not reported. The heterodisomy detection rate is not currently known for this assay.

**References**:

E1. Li H, Handsaker B, Wysoker A, Fennell T, Ruan J, Homer N, et al. The Sequence Alignment/Map format and SAMtools. Bioinforma Oxf Engl. 2009 Aug 15;25(16):2078–9.

E2. Paila U, Chapman BA, Kirchner R, Quinlan AR. GEMINI: integrative exploration of genetic variation and genome annotations. PLoS Comput Biol. 2013;9(7):e1003153.

E3. Chang CC, Chow CC, Tellier LC, Vattikuti S, Purcell SM, Lee JJ. Second-generation PLINK: rising to the challenge of larger and richer datasets. GigaScience. 2015;4:7.

E4. Danecek P, Auton A, Abecasis G, Albers CA, Banks E, DePristo MA, et al. The variant call format and VCFtools. Bioinforma Oxf Engl. 2011 Aug 1;27(15):2156–8.

E5. Quinlan AR, Hall IM. BEDTools: a flexible suite of utilities for comparing genomic features. Bioinforma Oxf Engl. 2010 Mar 15;26(6):841–2.
